# Toward resilient cities: Mapping the interconnected factors shaping urbanization in a dual analysis framework

**DOI:** 10.1371/journal.pone.0317342

**Published:** 2025-06-06

**Authors:** Naveed Ahmed, Wenlong Lou, Jameel Ahmed, Ali Akbar

**Affiliations:** 1 School of Humanities and Law (School of Public Administration), Yanshan University, Qinhuangdao, Hebei, P. R. China; 2 Beijing-Tianjin-Hebei Cooperative Development Management Innovation Research Centre, the Key Research Base of Humanities and Social Sciences in Higher Education Institutions of Hebei Province, Qinhuangdao, P. R. China; 3 Department of Commerce, University of Balochistan, Balochistan, Quetta, Pakistan; 4 Department of Statistics and Finance, School of Management, University of Science and Technology of China, Anhui, Hefei, P. R. China; Tallinn University of Technology School of Engineering: Tallinna Tehnikaulikool Inseneriteaduskond, ESTONIA

## Abstract

Utilizing the Push and Pull theory, this study examines the impact of socio-economic disparities and natural disasters on migration and urbanization. With a global significance, the shift of population from rural to urban areas carries profound implications for societies and economies. In the specific context of Pakistan, the research delves into the driving forces behind the rapid urbanization in Karachi and Quetta. Employing a mixed-methods approach, combining quantitative data and qualitative Geographic Information System (GIS) analysis, the study surveyed 1120 migrants. Results indicate a significant positive correlation between socio-economic disparities, natural disasters, and migration, highlighting the interplay of rural push factors and urban pull factors. GIS and satellite images reveal noticeable expansion in covered areas in both cities. The study underscores the importance of effective disaster management and resilient infrastructure to mitigate the impact of natural disasters on migration and urbanization. The findings offer valuable insights for policymakers and academics, discussed in the later sections of the study.

## 1. Introduction

Urbanization in the developing world has undergone a remarkable and dynamic transformation [1,2]. Between 1950 and 2018, the urban population in less developed regions experienced a substantial increase, surging from approximately 0.3 billion to 3.23 billion. During this period, the proportion of the urban population more than doubled, soaring from 18% to 51% [3]. The challenges faced by developing countries during urbanization are often exacerbated by significant rural-urban migration—a notable distinction between developing and developed nations [4–6]. This movement is often driven by socio-economic inequalities and natural disasters that displace populations, leading to urban migration [4,7–9]. Together, these factors highlight the complex and multifaceted drivers of rapid urbanization. [10].

**Fig 1:**
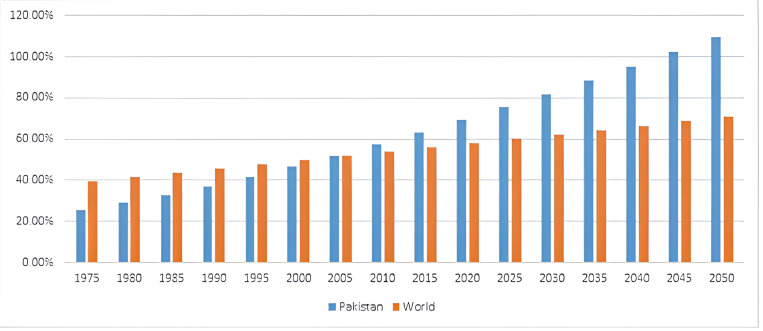
Urbanization rate in Pakistan and the world, 1975-2050. Source: [20].

In recent decades, Pakistan has experienced a remarkable upswing in urbanization, with projections indicating a trajectory towards 60% urban population by 2050 [3]. This rapid urbanization contributes to global integration, fostering economic exchange, international trade, and cultural diversity [11,12]. Pakistan is one of the world’s most diverse countries in terms of its culture and geographical location [13]. After the outbreak COVID-19 and the flood of 2022 Pakistan is caught in the spiral of these calamities in the shape of climate change, natural disasters and economic disparities [11]. All these factors immensely affected this country in the last few years now [14]. Due to such natural calamities and economic disparities the migration towards the major cities has been increased manifold, especially in the provinces of Sindh and Balochistan [15]. Sindh is the second most populated province of Pakistan, while Balochistan is the least populated [16]. These two provinces are equally affected by economic disparities and natural disasters like drought, floods and earthquake [13]. These factors further lead to the migration of people toward the capital cities of these provinces, namely Karachi and Quetta [17]. Karachi is the economic hub of Pakistan and is considered to be metropolitan city of the province where there are more opportunities for employment [18]. Meanwhile, Quetta is the only major city in its province offering a better standard of living compared to other parts of the region [19]. Fig 1 illustrates the trajectory of urbanization in Pakistan compared to global trends from 1975 to 2050, providing a visual representation of the nation’s swift urbanization.

Pakistan’s urbanization significantly boosts global interconnectedness in various ways (S1 Fig). Firstly, the rapid urban growth and increasing affluence create a thriving market for goods and services, fostering globalization [21–23]. Secondly, it enhances integration into international trade and investment [24,25], driven by the growing middle class demanding foreign products and services, stimulating economic exchange. Thirdly, urbanization promotes pronounced cultural exchange making cities like Karachi increasingly cosmopolitan and diverse due to rural and international migration [23,26,27].

**Fig 2:**
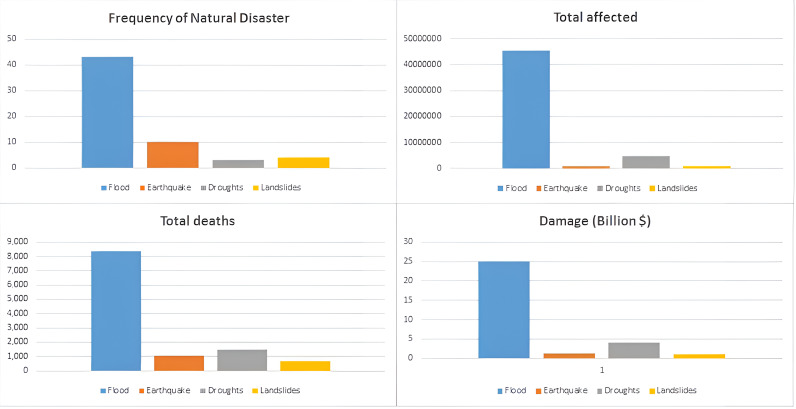
Snapshot of natural disasters in last 10 years. Source: [47].

Rural-urban migration theories view migration as the result of a combination of push and pull factors. Push factors (making people to leave their homes) [28,29], originating from rural areas, such as socio-economic disparities shape urbanization, evident in the uneven distribution of wealth, resources, and opportunities between urban and rural regions [30–32]. Disparities include limited access to quality education [33], healthcare [34], and employment opportunities [35], coupled with lower living standards [36,37]. Moreover, a large number of people are affected by natural disasters such as floods [38], earthquakes [39,40], and droughts in the past decade that have significantly pushed the urbanization in Pakistan (See Fig 2). These calamities often devastate rural communities, leading to loss of livelihoods, infrastructure, and homes resulting population displacement, forcing individuals to seek refuge in urban areas [36,37]. The influx of migrants from disaster-affected regions further contributes to the rapid urbanization observed in the country [21,27].

While pull factors (drag people to another place) [28,29], drawing people towards urban centers, such as improved living conditions [34,41,42], greater access to education [43], healthcare [35,38,44], employment, and higher wages [45,46]. This interplay of factors creates a dynamic where certain factors push people towards urban areas while others pull them, underscoring the intricate nature of the urbanization process. Pakistan’s urban-rural income gap (URIG) perpetuates division and fosters rural-to-urban migration [30,31].

The swift surge of urbanization in Pakistan stems from socio-economic inequalities, natural calamities, and rural-urban migration. However, varying conclusions from multiple investigations hinder consensus on the primary driving forces. Urbanization has the potential to alleviate socio-economic imbalances by shifting labor from rural to urban sectors, leading to heightened productivity, job expansion, and improved living standards [48–50]. Studies support this notion, showing urbanization reduces income disparities and poverty [51,52]. For instance, Ul-Huda et al. [53] found urbanization in Pakistan decreased income gaps particularly in major cities like Karachi and Lahore. Afzal et al. [48] in 2018 noted its positive impact on employment and poverty reduction. However, the benefits of urbanization are often undermined by discriminatory institutional arrangements and unequal development policies. Factors like rural-urban infrastructure divide and limited rural access to health and education widen disparities [24,54,55]. Unequal resource allocation and urban-focused investments tend to deepen socio-economic gaps, leaving rural areas further behind [56–58]. Understanding the interplay of socio-economic disparities and the challenges posed by natural disasters—such as flooding and droughts—is crucial for formulating effective policies to guide urbanization and address these growing inequalities [30–32].

Exploring the relationship between these factors within the Push and Pull theory framework is vital for a nuanced understanding of rural to urban population movements and urbanization patterns in Karachi and Quetta. While previous studies have explored various aspects of urbanization and migration in Pakistan, a gap exists in understanding the specific influence of socio-economic disparities and natural disasters on rural to urban migration in these specific cities [10,59]. Previous studies have examined these factors individually discrepancies in data sources, measurement indicators, and analytical methods have further challenged the formulation of evidence-based policies. A holistic review is necessary to guide effective interventions.

This study combines quantitative data on socio-economic disparities, natural disasters, and rural to urban migration with qualitative GIS analysis to highlight urbanization trends and landcover changes in Karachi and Quetta. Socio-economic disparities and natural disasters are not the sole migration drivers in Pakistan. Refugees from Afghanistan due to war and internally displaced persons (IDPs) also significantly contribute, especially in Khyber Pakhtunkhwa (KPK). While recognizing the broader context of migration, this research specifically examines the impact of socio-economic disparities and natural disasters on rural to urban migration patterns, ultimately affecting urbanization in Karachi and Quetta. The findings aim to inform evidence-based policymaking and urban planning, not only in Pakistan but also in other developing countries facing similar challenges. Understanding these dynamics can lead to targeted strategies for inclusive and sustainable urban development aligned with the 2030 Agenda for Sustainable Development [60–62].

The study addresses gap by setting following objectives and research questions:

1. How do socio-economic disparities, such as income inequality, education, and healthcare access, influence migration patterns from rural to urban areas in Karachi and Quetta?
2. To what extent do natural disasters, such as floods and earthquakes, contribute to migration flows from rural to urban areas in the Karachi and Quetta?
3. How does migration from rural areas to Karachi and Quetta impact urban growth patterns, infrastructure development, and social services in these cities?

Overall, the study aims to provide insights into the complex dynamics of socio-economic disparities, natural disasters, migration, and urbanization, enhancing understanding of urban development processes.

## 2. Theoretical background and literature review

### 2.1. Theoretical background

Most of the literature on urbanization argues that urbanization is closely linked to migration. When rural workers move to cities, urbanization leads to knowledge exchange, innovation, and productivity growth, creating a positive feedback loop between city size and productivity [63]. Migration has become a highly complex dilemma in the development process. One of the most important works on rural-urban migration theories was done by Ravenstein, who viewed migration as the result of a combination of push factors, (making people to leave their homes), and pull factors, (drag people to another place) [28,29]. The “push and pull” theory is a widely recognized framework that helps explain the factors influencing migration patterns from rural to urban areas. It suggests that people are pushed out of their place of origin due to negative factors and pulled toward their destination by positive factors. The pull factors are characterized by the economic and environmental factors that lead the people to attract (Pull) from the place of origin to the place of destination. On the other hand push factors include less job opportunities, political unrest, and losses [64]. Migration thus can be considered as a shrewd decision by individuals to lessen socio-economic disparities and environmental factors such as natural disasters that can be more in their place of origin.

The factors propelling migration encompass conditions that compel individuals to depart from their residences. These can be classified as push factors, encompassing socio-economic disparities and the occurrence of natural disasters, which contribute significantly to the surge in rural-to-urban migration. These push factors are characterized by subpar living standards, restricted job, educational, and healthcare opportunities, as well as the prevalence of natural disasters in rural regions. These factors compel individuals to abandon their homes in search of improved prospects elsewhere [35,38,44]. Concurrently, the allure of urban areas, represented as pull factors, including enhanced access to education, healthcare, employment possibilities, and an elevated quality of life, serves as motivation for migrants to relocate to cities [34,41,42]. Consequently, urbanization experiences a notable upswing as an increasing number of individuals migrate from rural to urban areas in pursuit of enhanced life prospects.

### 2.2. Literature review

Regarding the importance of push and pull factors as a driving force for migration, many studies have developed different views and aspects of these factors. Such as, access to quality education [33], healthcare [34], employment opportunities [35], better career prospects [65], higher wage, and resilient infrastructure compel individuals migrate towards urban areas [45,46]. The authors state that limited accessibility to these socio-economic factors in rural areas is of the contributing factors that push migrants; to overcome these disparities migrants are pulled towards urban areas.;  to overcome these disparities, migrants are pulled towards urban areas

Urbanization is a complex and dynamic process influenced by a numerous push and pull factors as a driving force for migration, including socio-economic disparities and natural disasters. Understanding the interplay of these factors and their impact on rural-to-urban migration is crucial for effective policy development and urban planning. Despite numerous studies on aspects of urbanization and migration in Pakistan, there remains a significant research gap concerning the specific influence of socio-economic disparities and natural disasters on rural-to-urban migration in Karachi and Quetta.

#### 2.2.1. Socio-economic disparities.

The decision to migrate is often driven by a combination of “push” and “pull” factors. Push factors, such as limited economic prospects, inadequate education, healthcare, and infrastructure in rural areas, compel individuals to seek better opportunities in urban centers. Many studies have developed different views and aspects of these factors. Such as, access to quality education [33], healthcare [34], employment opportunities [35], better career prospects [65], higher wage, and resilient infrastructure compel individuals migrate towards urban areas [45,46]. On the other hand, urban areas act as magnets, pulling migrants with the promise of higher wages and improved living conditions [45,46]. The authors state that limited accessibility to these socio-economic factors between rural areas is of the contributing factors that push migrants and to overcome these disparities migrants are pulled towards urban areas.

Migration can have mixed effects on socio-economic disparities. Some studies examined the impact and relationship between the migration and the living standards in the place of current residence. Lipton [66], argued that internal remittances also worsen rural inequalities in India because they are mainly from high-income villagers. Evidence from Mali and Senegal also suggests that remittances cause rural households to reduce their work effort, thereby reducing the effectiveness of migration as an instrument for poverty reduction [67].

Moreover, evidence from Mexico indicates that remittances from internal and international migrants have an egalitarian impact on rural income distribution [68]. Recent studies from Thailand and Vietnam also showed that migration reduced socio-economic disparities through a balanced distribution of productive assets [69], and had a positive impact on socio-economic status [70]. Studies that analyzed the impact of migration on the well-being of the migrant households in the destination places concluded that the degree of success of migrant workers depends on human and social capital [71,72], the length of the migration period, the quality of working conditions, and the existence of social networks [73]. Examining these effects is essential to determine how migration influences socio-economic disparities. Pakistani cities are facing a number of challenges, including climate change, haphazard expansion, and housing shortage.

#### 2.2.2. Natural disasters.

The impact of natural disasters can lead to population migration, with individuals being pushed to move due to the effects of the disasters and being pulled towards cities. Natural disasters, including floods, earthquakes, and droughts, can significantly influence migration patterns. Some researchers investigated relationship between ecological factors and migration. The authors state that the occurrence and severity of natural disasters can influence migration decisions [10,59]. Particularly, rural communities are vulnerable to the devastating consequences of events, ranging from floods and droughts to earthquakes [38]. These calamities often result in the loss of livelihoods, the destruction of homes, and the disruption of vital infrastructure [39,40]. Faced with the aftermath of a natural disaster, individuals find themselves compelled to seek safety, support, and the means to rebuild their lives in urban centers [38]. These occurrences have pushed individuals away from disaster-affected regions while pulling them toward urban areas with more resources [10,59]. Natural disasters add another layer of complexity to migration patterns, understanding the role of natural disasters in migration is crucial for effective urban planning.

#### 2.2.3. Migration.

Migration serves as a pivotal response to the interplay of socio-economic disparities and the impacts of natural disasters. The decision to migrate is often influenced by a complex web of factors, including socio-economic disparities and the occurrence of natural disasters, reflecting a dynamic relationship [74,75]. Socio-economic disparities act as significant “push” factors, compelling individuals to migrate from economically disadvantaged regions to urban centers [28,64], (see “Socio-economic Disparities” section). In addition to socio-economic disparities, the occurrence and severity of natural disasters can significantly influence migration patterns. Individuals and communities affected by natural disasters are often pushed to seek safety, support, and resources available in urban areas, making urban areas act as pull factors for disaster-affected populations [76], (see “Natural Disasters” section).

Rural to urban migration is a significant phenomenon in developing countries, driven by factors such as better job opportunities, career prospects, higher wages, and access to social benefits in cities [43]. It is also influenced by agricultural land scarcity in rural areas [77]. Migration is work-based, as migrants provide the necessary labor force for urban production, leading to increased aggregate output and economic growth [78]. Education-based migration is also important, as cities offer high-quality education institutions and opportunities for human capital accumulation [79]. Rural-urban migration has wide impacts on growth, income distribution, and poverty alleviation [80].

The complex interplay between socio-economic disparities and natural disasters further shapes migration patterns. In the context of the present study, examining the role of migration in response to socio-economic disparities and natural disasters is essential for comprehending the dynamics of urbanization, population movement, and resilience [81]. Understanding the specific factors that drive migration in these contexts is crucial for policymakers and urban planners seeking to develop effective strategies for sustainable urban development and disaster mitigation [38,82].

In theory, successful migration over several years should positively impact the rural-urban income gap. Despite Pakistan being the most urbanized nation in South Asia with 36 percent of the population residing in cities [30], there still exists a significant rural-urban divide in terms of socio-economic disparities, with rural disparities being notably higher than urban across all regions [83]. While previous studies have explored aspects of migration and urbanization. Such as, income inequality and migration [30], socio-economic determinants of migration [84], rural–urban migration improve employment and household welfare [49]. Others have explored aspects of urban resilience, community engagement [36,85,86], challenges of urbanization, such as climate change, haphazard urban expansion, and a lack of basic amenities [87,88], urbanization and governance perspective [21], urbanization and political change [27], urbanization and local governance challenges [54], and urban-rural differentials of health and educational inequality [89]. All mentioned studies have predominantly utilized primary data from population censuses, relative measure index, labor force surveys, and existing secondary scholarly literature.

To the best of authors' knowledge none of the studies have explored the interesting interplay of the socio-economic disparities, natural disasters and their impact on migration and urbanization. This study addresses this gap in the literature by examining the impact of socio-economic disparities and natural disasters on rural-to-urban migration in Karachi and Quetta. By doing so, we aim to provide a holistic view of the forces shaping migration and urbanization trends in Pakistan, offering insights that can inform policy and development initiatives in both urban and rural areas.

The existing literature provides valuable insights into the role of socio-economic disparities and natural disasters in driving rural-to-urban migration. However, all mentioned studies either have predominantly utilized primary data from 1998 population censuses, or relative measure index, labor force surveys, and existing secondary scholarly literature. A research gap remains regarding the specific influence of these factors on urbanization in Pakistan, especially in the cities of Karachi and Quetta. While previous studies have explored aspects of urbanization and migration, none have explicitly investigated the major factors contributing to the rapid growth of urbanization in these areas. The lack of recent and comprehensive data, given that Pakistan’s last population census was conducted in 1998, also presents potential inaccuracies in the understanding of these trends. Therefore, this study aims to bridge this gap by examining the interplay of socio-economic disparities and natural disasters and rural-to-urban migration on urbanization in Karachi and Quetta. By doing so, we aim to provide a holistic view of the forces shaping migration and urbanization trends in Pakistan, offering insights that can inform policy and development initiatives in both urban and rural areas.

The motivation for this study is the lack of studies on understanding the major factors contributing to rapidly growing urbanization in Pakistan. Our study in this regard is the first in its nature taking into account both quantitative and qualitative analysis. Filling this gap in the literature requires a robust analysis of these factors which may contribute in the process of migration. We particularly examined and tested empirically the interplay between socio-economic disparities, natural disasters and rural to urban migration, as well as analyzed urban land growth (urbanization) through GIS. This study uses a unique dataset of migrant in urban areas. It aims to provide new insights into the role of migration due to socio-economic disparities and natural disasters contributing in rapid urbanization in Sindh and Balochistan, Pakistan focusing on the Push and Pull theory of migration.

**Fig 3:**
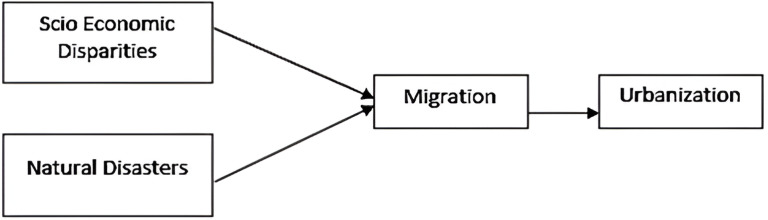
Conceptual framework.

Based on theoretical framework and an extensive literature, present study hypothesizes and proposes a conceptual framework Fig 3. We believe that socio-economic disparities, natural disasters lead migration and migration in different areas lead urbanization.

- **H1.** Socio-economic disparities will be positively related to rural to urban migration.
- **H2.** Natural disasters will be positively related to rural to urban migration.
- **H3.** Rural to urban migration will positively influence urbanization patterns in Karachi and Quetta, providing a contextual understanding of this relationship within the socio-economic and environmental conditions of Pakistan.

## 3. Materials and methods

### 3.1. Study area

This study focuses on the two provincial capitals of Pakistan: Karachi and Quetta. Karachi, located at 24.8600°N, 67.0100°E, is the largest city in Pakistan and spans a total area of 3,530 km². As the economic hub of the country, Karachi attracts significant internal migration due to its employment opportunities and urban infrastructure [15]. In contrast, Quetta, situated at 30.1796°N, 66.9750°E, covers an area of 2,653 km² and serves as the administrative and economic center of Balochistan. Despite its smaller size, Quetta experiences notable migration flows, driven by its relatively better living standards and services compared to other parts of the province [16].

**Fig 4:**
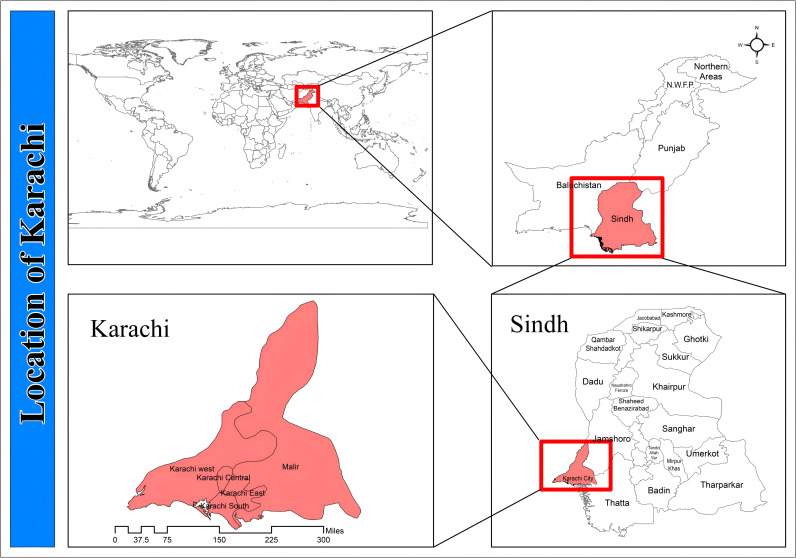
Location map of study area. Source: Basemap from admin 2 level shapefiles, district boundaries of Pakistan, DIVA-GIS (https://www.diva-gis.org/).

Both cities were selected as focal points due to their strategic importance, distinct urban characteristics, and their prominence as destinations for rural to urban migration influenced by socio-economic disparities and natural disasters [13]. Figure 4 illustrates the geographical location of the study area.

### 3.2. Methodology

#### 3.2.1. Sample and data collection.

In order to get accurate responses, the researcher visited designated sites multiple times to assess questionnaire difficulty levels before formal data collection. To get more robust responses for our survey we translated our questionnaire into national language (Urdu), using the traditional back-translation technique from English to ensure accuracy [90], and feedback from 50 migrants helped refine unclear items. No major changes were made after this preliminary observation. Due to the nature of the study data was collected through primary as well as secondary source. In the first stage of the study primary data was collected on 5-point Likert scale from a sample of N = 1120 respondents residing in the two targeted cities through an adapted survey questionnaire. The questionnaire was divided into demographic section (age, gender, education, marital status, occupation, city of residence and years of residency in current city), questions related to socio economic disparities, natural disaster and rural to urban migration. Informed written consent was obtained from all participants, following local laws and ethical guidelines outlined in the Declaration of Helsinki.

The sample size of 1,120 migrants was determined based on the study’s focus on migration patterns and challenges, ensuring adequate representation of the targeted population while maintaining statistical rigor. We followed Haier’s minimum sample size criteria, requiring at least 100 respondents for models with five or fewer variables, with at least three items per construct and item communalities of no less than 0.6 [91]. The chosen sample meets these criteria and supports robust data analysis. While including local residents could provide a comparative perspective, this study focused exclusively on migrants to address their unique socio-economic and environmental challenges. Future studies could explore a broader population for comparative insights. In second stage secondary data was collected to analyze urbanization and landcover change. The secondary data was collected from Survey of Pakistan (SOP) topo-sheets of Karachi and Quetta at a scale of 1:50,000. Additionally, satellite imagery from Thematic Mapper (TM) and Enhanced Thematic Mapper Plus (ETM+) sensors was utilized. An administrative boundary map was also incorporated as a data source.

To enhance reliability and objectivity, we used primary data collected via survey questionnaires for analysis and hypothesis testing. Our study required assessing both primary and secondary data to fully explore the research topic. Primary data offers tailored insights, while secondary data provides historical context, completeness, and efficiency. This dual approach ensures a comprehensive understanding of migration and urbanization dynamics. Supplementing our analysis with GIS-derived secondary data allows for a focused investigation into socio-economic and natural disaster drivers. This integrated approach enables a nuanced understanding of urbanization trends. Through this combined method, we aim to offer a comprehensive insight into the complexities of our research.

In research, blending primary and secondary data through mixed methods is widely used to enhance depth and credibility. For example, a study on climate change and migration in Bangladesh utilized household surveys, focus groups, key informant interviews, and academic literature [92]. Similarly, research on healthcare choices in Singapore’s low-income urban community combined surveys, interviews, focus groups, and secondary data analysis (85). Another study on transnational migration showcased the effectiveness of mixed methods [93,94]. These examples underscore the value of integrating diverse data sources for a comprehensive understanding of complex research topics.

#### 3.2.2. Respondent-driven sampling (RDS).

The RDS method, a type of snowball sampling, was employed for primary data collection. This method involved peer-to-peer recruitment within social networks, where initial participants (seeds) referred additional respondents from their network of friends [95]. The iterative recruitment process continued until the desired sample size was achieved. RDS has been used to access hard-to-reach populations and provides unbiased estimates under certain conditions [96,97]. It approximates random sampling methods and allows for generalization of findings to the target population [98]. RDS has been used in various fields, including social psychology research in Indonesia to explore sensitive social issues among hidden or hard-to-reach sub-population [99]. However, the accuracy of RDS in estimating certain network features, such as homophily, remains unclear. Overall, RDS is a valuable method for sampling hard-to-reach populations and has been widely used in various research fields.

#### 3.2.3. Measures.

The study employed an adaptive survey method to collect data on variables such as natural disasters, socio-economic disparities, and rural to urban migration. Survey responses captured individual experiences with socio-economic disparities (e.g., access to roads, healthcare, electricity, jobs) and natural disasters (e.g., floods, earthquakes) in their place of origin and current residence. These variables were assessed using a 5-point Likert scale and statistically analyzed to identify key disparities influencing migration. This primary data provided tailored insights into the socio-economic, natural disasters and conditions driving migration patterns. Data were collected from respondents before and after migration to Karachi and Quetta, enabling the identification of push and pull factors associated with urbanization. The questionnaire, detailed in Table 1, utilized a 5-point Likert scale ranging from 1 = very poor and 5 = Very good to assess the importance of each factor. Adjustments to the questionnaire were made based on participant feedback. Ethical considerations were prioritized to ensure participant confidentiality, privacy, and safety.

**Table 1:** Measures of the constructs.

Category	Construct	References
Socio-economic Disparities	Family size	[100]
	Individual and family earnings	[101,102],
	Employment Opportunities	[103] [100] [103] [100] [103] [100] [103]
		Availability of Roads
		Availability of Transportation
		Availability of School
		Availability of Hospitals
		Availability of Electricity
		Availability of Communication signals
	Availability of Tap water	[103] [100] [100]
		Availability of Gas (Cooking Fuel)
		Availability of jobs
Natural Disasters	Flooding	[104]
	Earthquakes	
	Drought or water scarcity	
	Landslides	
Migration	Lack of employment opportunities	[103]
	Limited access to quality education	
	Insufficient healthcare facilities	
	Limited infrastructure development	
	Poverty or inequality	[104] [103] [104] [104] [104] [103]
		Lack of basic amenities (water, electricity, sanitation)
		Flooding
		Earthquakes
		Drought or water scarcity
		Landslides

#### 3.2.4. GIS- Urban growth and landcover change.

For urban growth and landcover analysis, secondary data including GIS and satellite imagery from the Survey of Pakistan (SOP) was utilized to generate administrative boundary map, landcover changes and urban growth trends in Karachi and Quetta. These spatial data points provided validation for the survey findings, specifically regarding infrastructure development and settlement expansion in the cities.

Satellite imagery from TM and ETM+ sensors, obtained from www.earthexplorer.usgs.gov, was processed to identify land cover types and urbanization trends. Images from 1992, 2002, 2013, and 2022 underwent atmospheric correction using the improved dark objects subtraction method, with water bodies serving as reference points. The corrected images were digitally enhanced for subsequent analysis.

#### 3.2.5. Controlled variables.

The demographic variables including age, education, gender, marital status and occupation were controlled for this study. In the past researches these variable shown significant effect on migration. See Table 5.

### 3.3. Data analysis

#### 3.3.1. Primary data analysis.

The collected survey data underwent comprehensive analysis using SPSS v.26 to uncover the drivers of urbanization in Pakistan. Descriptive statistics, such as mean, median, and frequency distributions, summarized the data, while correlation analysis explored relationships between socio-economic disparities, natural disasters, and migration.. Regression analysis examined the impact of these factors on urbanization, with demographic variables controlled to isolate key effects. Factor analysis was conducted to ensure construct reliability, as shown in Table 4. These analyses provided a detailed understanding of how socio-economic disparities and natural disasters influenced rural to urban migration patterns.

#### 3.3.2. Spatial data analysis.

The analysis of satellite imagery and land cover changes was conducted using ArcMap 10.8. The processed images were classified into four land use classes: Settlement, Barren Land, Vegetation, and Water Bodies. GIS techniques and satellite data were employed to map land cover changes and urban growth trends in Karachi and Quetta. These spatial datasets provided validation for survey findings, particularly in identifying infrastructure development and settlement expansion. Advanced spatial analysis techniques, including overlay analysis, buffer analysis, and change detection, were applied to pinpoint areas of rapid urban growth and significant land cover changes. By integrating quantitative survey data with spatial analysis, the study presented a comprehensive perspective on urbanization dynamics. This combined approach highlighted the primary factors driving urban growth and the corresponding transformations in land use patterns in Karachi and Quetta.

## 4. Analysis and results

### 4.1. Socio-economic disparities, natural disasters and migration

The study focuses on understanding the major factors contributing to rapidly growing urbanization in Pakistan. It explores the relationship between socio-economic problems, natural disasters, rural to urban migration, and the subsequent increase in urbanization. The data for the study was collected from migrants living in cities, and it compares the perceptions of migrants regarding various factors before and after their migration.

Table 2 presents the demographic characteristics of the study population in order to provide a comprehensive overview of the sample population, providing crucial insights into the socio-demographic context of the study. Table displays the demographics of the migrant target population (N = 1120), categorized into seven major sections: gender, marital status, occupation, age, education level, current city of residency, and years of residence in the current city. The sample comprises 574 (51.2%) males and 546 (48.8%) females, representing a gender-balanced population.

**Table 2:** Demographics of controlled variables.

Controlled variables	Demographics of controlled variables	Frequency	Percentage
Gender	Male	574	51.2
	Female	546	48.8
Marital status	Married	560	50
	Unmarried	515	46
	Widow	33	2.9
	Divorced	12	1.1
Occupation	Govt.employee	283	25.3
	Pvt.employee	274	24.5
	Business	344	30.7
	Unemployed	219	19.6
Age in years	18-25	94	8.4
	25-30	116	10.4
	30-35	285	23.7
	35-40	184	16.4
	40 and Above	461	41.2
Education	Primary	103	9.2
	Secondary	280	25
	Higher Secondary	281	25.1
	Bachelors	275	24.6
	Master and Above	181	16.2
City of Residency	Karachi	685	61.2
	Quetta	435	38.8
Years of Residency in Current city	1-2	241	19.1
	2-3	174	15.5
	3-4	210	18.8
	4-5	329	29.4
	5 or more	193	17.2

*N = 1120*

Regarding marital status, half of the respondents (50%) were married, while 515 (46%) were unmarried. Only a small proportion were widowed (2.9%) or divorced (1.1%). Most participants were aged 40 years or older (41.2%), followed by 30–35 years (23.7%) and 35–40 years (16.4%). In terms of employment, 30.7% were engaged in business, 25.3% were government employees, and 24.5% were employed in the private sector, with 19.6% being unemployed. Educationally, respondents were almost evenly distributed across higher secondary (25.1%), secondary (25%), and bachelor’s education levels (24.6%), with smaller groups having primary education (9.2%) or master’s and above (16.2%).

The majority of participants resided in Karachi (61.2%), with the rest in Quetta (38.8%). Additionally, 29.4% had lived in their current city for 4–5 years, and a notable proportion (19.1%) had only recently moved within the past 1–2 years.

The Table 3 presents descriptive statistics for three variables: Socio-Economic Disparities, Natural Disaster, and Rural to Urban Migration. Socio-Economic Disparities has a mean of 2.61, indicating a moderate perception of disparities, with a low standard deviation (0.357), suggesting consistency in responses. Natural Disaster Impact has a mean of 1.79, showing a low to moderate perception of impact, with a standard deviation of 0.288, indicating similar responses across the sample. Migration level has a mean of 2.81, reflecting moderate rural to urban migration levels, with a higher standard deviation (0.435), indicating greater variability in migration experiences.

**Table 3:** Descriptive analysis.

Variables	N	Min	Max	Mean (M)	SD
Socio-Economic Disparities	1120	1.44	5	2.6121	0.357
Natural Disaster Impact	1120	1	2.5	1.7897	0.288
Migration Level	1120	1.5	4.2	2.8133	0.435

The results in Table 4 indicate significant positive correlations between socio-economic disparities and natural disasters (r = 0.128, p < .01), between socio-economic disparities and rural to urban migration (r = 0.547, p < .01), and between natural disasters and migration (r = 0.189, p < .01). The correlation between socio-economic disparities and rural to urban migration (r = 0.547) is notably stronger compared to the other two relationships, indicating a more substantial association between socio-economic factors and migration patterns. The p-values for all correlations are less than 0.01, suggesting that the relationships observed are statistically significant. The 95% confidence intervals for all correlations—ranging from [0.089, 0.167] for socio-economic disparities and natural disasters, [0.512, 0.582] for socio-economic disparities and rural to urban migration, and [0.146, 0.232] for natural disasters and migration—further confirm the reliability of these findings.

**Table 4:** Correlations between socio-economic disparities, natural disasters, and migration.

Variables	N	r (Correlation Coefficient)	p-value	Confidence Interval (95%)
Socio-economic Disparities vs. Natural Disasters	1120	0.128	<0.01	[0.089, 0.167]
Socio-economic Disparities vs. Migration	1120	0.547	<0.01	[0.512, 0.582]
Natural Disasters vs. Migration	1120	0.189	<0.01	[0.146, 0.232]

To check the reliability of the scale used in our study we conducted exploratory factor analysis (EFA) Table 5. All the items demonstrated good factor loadings above the minimum threshold of 0.5, and they were appropriately loaded onto their respective scales. Additionally, the Cronbach’s Alphas for socio-economic disparities, natural disasters, and migration were found to be 0.932, 0.876, and 0.916, respectively. All of these values exceed the minimum criterion of 0.7, indicating high internal consistency and reliability of the scales.

**Table 5:** Exploratory factor analysis.

Constructs	Item No.	Loadings	Cronbach’s Alpha
Socio economic disparities	SC1	0.842	**0.932**
	SC2	0.834	
	SC3	0.812	
	SC4	0.873	
	SC5	0.789	
	SC6	0.882	
	SC7	0.763	
	SC8	0.761	
	SC9	0.821	
Natural Disaster	ND1	0.887	**0.876**
	ND2	0.863	
	ND3	0.855	
	ND4	0.853	
Migration	MG1	0.852	**0.916**
	MG2	0.836	
	MG3	0.834	
	MG4	0.792	
	MG5	0.752	
	MG6	0.881	
	MG7	0.873	
	MG8	0.868	
	MG9	0.859	
	MG10	0.784	

The hypotheses of the study were tested using SPSS linear regression, and the results are presented in Table 6. Hypothesis 1 aimed to examine the relationship between socio-economic disparities and migration. The findings revealed a significant positive effect of socio-economic disparities on rural to urban migration (β = 0.128, p < .001, LLCI = 0.035, ULCI = 0.247) at the 95% confidence interval, thus confirming hypothesis 1. The second hypothesis of the study aimed to investigate the impact of natural disasters on rural to urban migration. The results indicate a significant positive association between natural disasters and rural to urban migration (β = 0.09, p < .001, LLCI = 0.047, ULCI = 0.223), supporting hypothesis 2. Moreover, the lower level confidence interval (LLCI) and upper level confidence interval (ULCI) in both the cases do not contain zero, which provides further support to our testable statements.

**Table 6:** Regression analysis.

Relationship	β	T	Sig.	R²	F	Sig.	Hypotheses
H1: SC => MIG	0.128	2.607	0.000	0.016	6.796	0.000	Accepted
H2: ND => MIG	.0.090	3.005	0.000	0.008	9.029	0.000	Accepted

*SC =* Socio economic disparities, *ND =* Natural disaster, *MIG =* Migration. ***p <.001

Table 7 represents the results of the mass migration towards the two major cities. The data was analyzed on two prongs: before and after migration. The above results show that there is an increase in economic indicators in terms of earnings and the number of people per family. More specifically, from the results, we can say that the average or mean value of the earning of the number of people per family is increased after migration, e.g., 2.92 > 1.74. This shows that cities offer more job opportunities as compared to rural areas. As evident from the results, after migration, the average earning of the family also increased from 3.16 to 3.86. In other words, we can say there are more earning and employment opportunities in these cities. Therefore, migration has a positive effect on people’s average earnings.

**Table 7:** Economic impact of migration on family earnings.

Particulars	Options	Frequency	Percentage	Mean	SD
Family members	1-2	85	7.6	**3.43**	**1.18**
	3-4	158	14.1		
	5-6	299	26.7		
	7-8	340	30.4		
	9 or more	238	21.3		
People earning **BEFORE** migration	01	516	46.1	**1.74**	**0.94**
	02	496	44.3		
	03	33	2.9		
	04	31	2.8		
	05 or more	44	3.9		
People earning **AFTER** migration	01	291	26	**2.92**	**1.55**
	02	227	20.3		
	03	182	16.3		
	04	116	10.4		
	05 or more	304	27.1		
Income in Rupees **BEFORE** migration	25000 or less	108	9.6	**3.16**	**1.2**
	25000-35000	219	19.6		
	35000-45000	362	32.3		
	45000-55000	237	21.2		
	55000 or more	194	17.3		
Income in Rupees **AFTER** migration	25000 or less	57	5.1	**3.86**	**1.57**
	25000-35000	39	3.5		
	35000-45000	237	21.2		
	45000-55000	494	44.1		
	55000 or more	293	26.1		

Respondents were asked to rate the socio-economic facilities and the frequency of natural disasters at the place of their origin and at their current residence. Table 8 represents the means and standard deviations (SD) of socio-economic disparities at the place of their origin and at the place of their current residence. The mean value for roads, transportation, schools, hospitals, electricity, and telecommunication signals is higher at the current residence as compared to the place of origin. It is evident from the results that migrants have better infrastructure and service facilities in place of their current residence (Urban areas), motivating them to move from rural to urban locations in search of better living conditions. Whereas, surprisingly, the mean ratings for drinking water and gas are lower at the current residence compared to the place of origin. This finding may indicate that access to clean drinking water and gas supply is relatively better in rural areas because of the lower population, which leads to concern about basic amenities in some urban regions.

**Table 8:** Socio-economic disparities and natural disasters at the place of origin and current residence.

Socio-economic disparities				
Item	Place of Origin		Current Residence	
	Mean	SD	Mean	SD
Roads	3.469	2.045	3.479	3.191
Transportation	2.658	0.9434	3.7027	3.17638
School	2.5875	0.9431	3.9063	3.163
Hospital	2.3938	1.024	3.7696	3.98865
Electricity	2.383	0.92945	3.5982	1.15284
Telecommunication signals	3.3661	1.3342	3.8304	0.69965
Drinking water	2.3652	1.02935	2.2761	1.1332
Gas	1.8848	1.04336	2.4161	1.19056
Jobs	2.417	1.28936	4.0277	1.00051
**Total**	**2.6121**	**0.35794**	**3.4453**	**0.81037**
**Natural disasters**				
**Items**	**Place of Origin**		**Current Residence**	
	**Mean**	**SD**	**Mean**	**SD**
Floods	2.2643	0.77596	1.0679	0.25161
Droughts	2.2071	0.71493	2.7223	0.91
Land sliding	1.0375	0.19007	1	0
Earthquakes	1.65	0.67298	1.6545	0.79615
**Total**	**1.7897**	**0.28865**	**1.6112**	**0.34593**

However, the most significant economic disparity is observed in job opportunities, with the mean value substantially higher at the current residence. This emphasizes that better employment prospects in urban areas act as a strong pull factor for migration, driving the influx of rural dwellers seeking better economic opportunities. The overall mean rating for socio-economic disparities is higher (3.4453) at the current residence as compared to the place of origin (2.6121), indicating that migrants perceive an overall improvement in their living conditions after migrating to urban areas.

Table 8 also represents the means and standard deviations of natural disasters at the place of origin and current residency. The findings of the study show that mean values at the place of current residence is less than the place of origin (1.6112) and (1.7897). Which indicates that there are less occurrences of natural disasters in the place of current residence. It further suggests that the people who migrated towards the cities perceived less risk of natural disasters Overall, the analysis of socio-economic disparities and natural disasters between the place of origin and the current residence discloses significant enhancements after migration. These findings support the notion that better socio-economic settings and strong infrastructure in cities initiate migration and add to the process of urbanization in Karachi and Quetta.

### 3.2. Urban growth and landcover change

**Fig 5:**
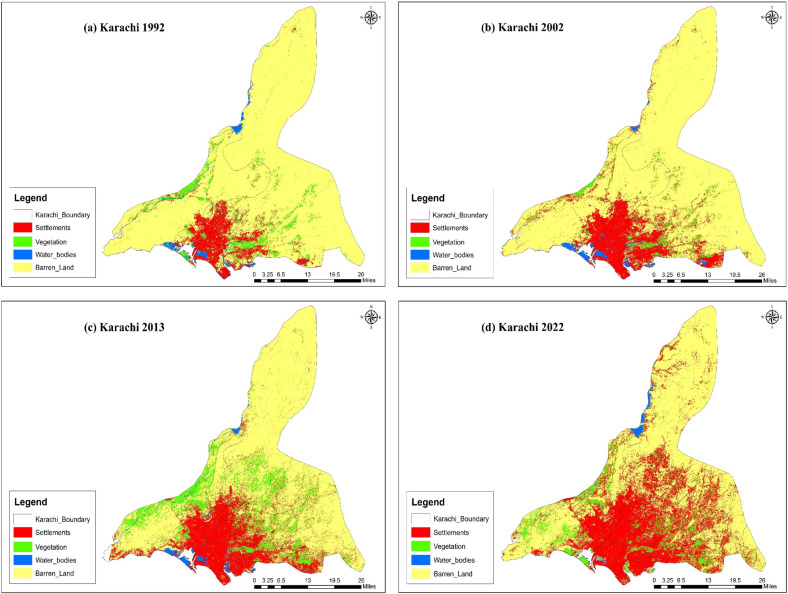
Land-use change in Karachi from (a) 1992, (b) 2002, (c) 2013 and (d) 2022. Source: Basemap from admin 2 level shapefiles, district boundaries of Pakistan, DIVA-GIS (https://www.diva-gis.org/).

**Fig 6:**
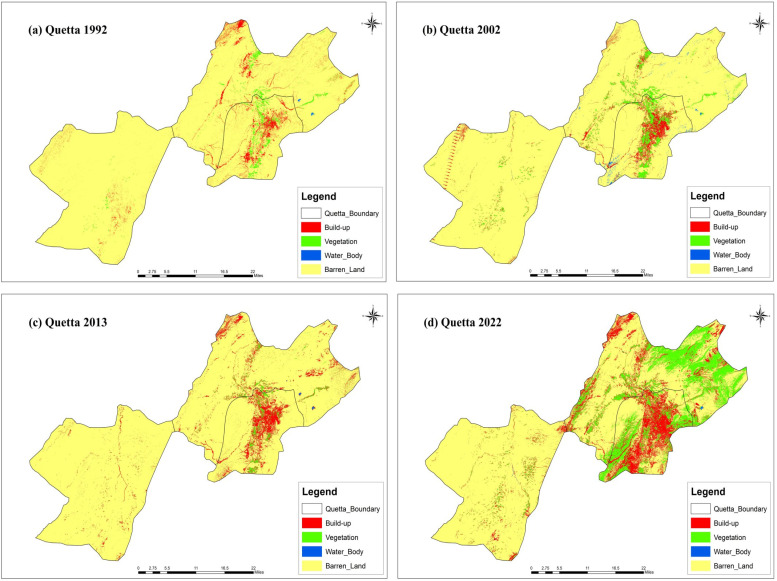
Land-use change in Quetta from (a) 1992, (b) 2002, (c) 2013 and (d) 2022. Source: Basemap from admin 2 level shapefiles, district boundaries of Pakistan, DIVA-GIS (https://www.diva-gis.org/). Fig 7 and ( S3 Fig) provide graphical information on the square kilometer areas of Karachi and Quetta, respectively. It is evident from the results that barren land in Quetta increased in 2022, whereas build-up area significantly decreased, as shown in Fig 6. A temporary increase in vegetation, particularly grass growth, which is visible in Figure 6 **(d)**. This change is largely due to the rainfall and flooding events, which is not a long-term change, but rather a short-term result of the floods. One of the reasons behind the increase in barren land and decrease in build-up area could be the 2022 flooding in Balochistan that killed 336 people [105].

The study was conducted in Karachi and Quetta, Pakistan, to check the urbanization. The results show that there has been a significant increase in urbanization. The classification of the satellite images into built-up and non-built-up areas for four temporal instants resulted in the creation of the land cover of Karachi and Quetta shown in Fig 5 and 6, which define the urban growth of specified times. The results show that over the period of time, the barren land decreased, whereas the built-up area increased significantly in both cities. Major urban growth occurred in the North-West of Karachi, towards Kemari Town and Gadap Town, while in Quetta, growth was observed in the North-West, South, and around Quetta Saddar.

**Fig 7:**
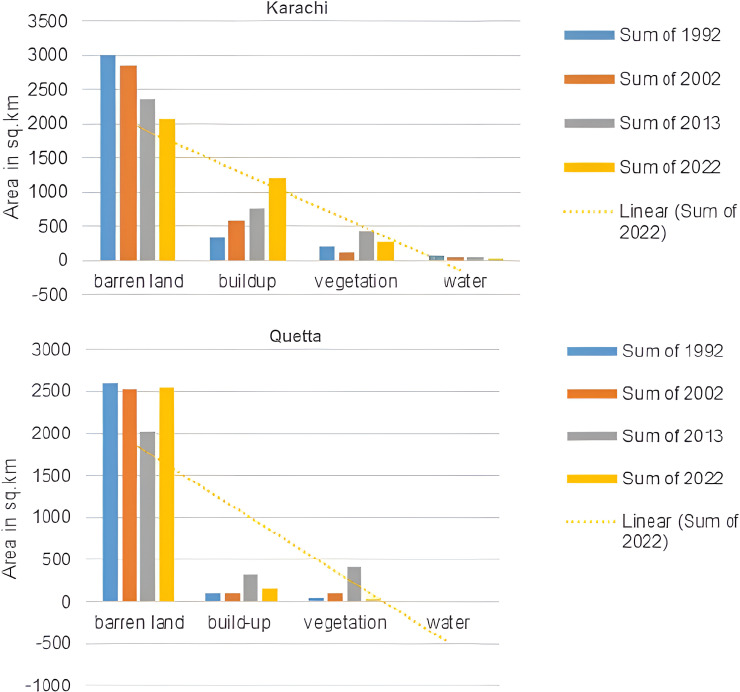
Area in sq. km of landcover of Karachi and Quetta city. Source: researcher.

Quetta was declared a disaster area due to the rains, and an emergency was declared in the province. In many cities of Balochistan, rainwater infiltrated many homes and made them uninhabitable, which pushed them to safer areas of Quetta. Around 10 million people were affected, as well as 241,659 houses that were either damaged or completely destroyed, and 1,230 km2 (304,000 acres) of crops were lost, along with an estimated half-million livestock killed [105]. On the other hand, Sindh province was the worst-affected province in the 2022 flooding. More than 14.5 million people were affected, over 1.8 million houses were either damaged or completely destroyed, and around half a million livestock were killed, along with 6,200 km2 (1,540,000 acres) of farmland swept away by the floods [105]. Most of the people from all over Sindh province were pushed by the 2022 disaster to big cities like Karachi.

## 4. Discussion

Our investigation into the factors influencing urbanization in Pakistan, framed by the push and pull theory, sheds light on the complex interplay of socio-economic disparities, natural disasters, and rural to urban migration. The economic rationale underlying our results can be elucidated through the lenses of human capital theory, environmental economics, and recent empirical studies. The study found that socio-economic disparities are greater in rural areas than in urban areas (see Table 8). This is supported by studies that have found that people living in rural areas are more likely to have limited access to basic services such as roads, transportation, electricity, better education, as well as health facilities, and be unemployed [34,106,107]. These socio-economic disparities are a major push factor for rural to urban migration, as people seek better opportunities in urban areas and eventually drive urbanization. Most young people look towards urban areas in order to get employment and quality of life [108,109], also observed in results where comparative household income increased and the number of people working in families increased after migration. This pattern of migration to urban areas in pursuit of improved living standards has been observed in various developing countries [38,71,73]. These findings resonate with migration patterns observed in other developing countries, such as Bangladesh and Indonesia, where natural disasters and socio-economic disparities have similarly acted as push factors for rural-to-urban migration [10,92]. Additionally, the regression analysis presented in Table 6 supported both hypotheses, demonstrating that socio-economic disparities and natural disasters have a significant positive association with migration, which eventually leads to increased urbanization.

Moreover, the study also found that natural disasters are more frequent in rural areas than in urban areas, which also act as pushing factors, compelling individuals to migrate to urban centers (see Table 8) and (S2 Fig). This is supported by studies that have found that 70% of natural disasters occur in rural areas [44]. Natural disasters disrupt livelihoods, devastate homes, crops, and businesses, and create an urgent need for relocation. In Pakistan, a significant number of people are forced to migrate each year due to the impacts of natural disasters. While exact figures vary, the trend highlights the vulnerability of rural and disaster-prone areas. This underscores that migration in these contexts is predominantly driven by “push” factors, such as environmental disruptions, rather than “pull” factors like urban opportunities. In line with existing research, these environmental push factors have led to a considerable influx of migrants into cities, making these cities attractive destinations due to their perceived resilience against such calamities [110]. Notably, the devastating floods in Balochistan and Sindh in 2022, supported by the spatial analysis presented in Fig 6 and the significant increase in barren land in 2022, served as major triggers for migration, exacerbating the process of urbanization in Karachi and Quetta. The steady growth of these cities can be partly attributed to the increasing number of people seeking safety and better prospects, transforming them into major urban centers in Pakistan.

The pronounced impact of socio-economic disparities as a push factor for rural to urban migration aligns with the principles of economic theories. In rural areas, limited access to basic services, including education and healthcare, creates a substantial barrier to human capital development [22,111]. As individuals seek better opportunities, particularly the youth aspiring for improved employment prospects and quality of life, migration to urban centers becomes an economically driven decision [4]. Our findings are consistent with recent studies [4,82,112,113], which highlight the pivotal role of human capital considerations in migration dynamics, supporting the assertion that migration often stems from a pursuit of enhanced economic opportunities.

The prevalence of natural disasters in rural areas acting as compelling push factors is economically rooted in the disruptions these disasters cause to livelihoods. The urgent need for relocation after the devastation of homes, crops, and businesses underscores the economic imperative for rural to urban migration. Recent studies, such as [4,44,92], reinforce our findings by emphasizing the economic consequences of natural disasters as significant drivers of migration. This aligns with the economic principle of minimizing risk and maximizing utility, as individuals migrate to urban areas perceived to be more resilient against calamities.

The spatial analysis, particularly illustrated in Fig 6, provides empirical evidence of the impact of natural disasters, such as the devastating floods in Balochistan and Sindh in 2022. The observed increase in barren land in 2022 serves as a tangible indicator of the economic repercussions of these events, triggering mass migration and exacerbating the urbanization process. Recent events and spatial data, as presented, offer real-world substantiation to our economic arguments, showcasing the relevance of our study in the contemporary context.

In terms of sustainable development, addressing socio-economic disparities can contribute to creating more inclusive societies, supporting SDG Goal 10 (Reduced Inequalities). By improving access to basic services, such as education, healthcare, and employment opportunities, governments can reduce the economic push factors that drive migration. This is aligned with the broader goal of fostering equality and enhancing quality of life for all citizens, which is integral to achieving long-term sustainability.

Furthermore, the connection between natural disasters and migration highlights the need for sustainable urban planning, which supports SDG Goal 11 (Sustainable Cities and Communities). Ensuring that cities are resilient to environmental shocks and investing in climate adaptation measures can mitigate the impacts of migration caused by disasters, leading to more sustainable and resilient urban environments.

Our study contributes significantly to the existing literature by not only identifying push and pull factors but also providing tangible policy implications. This approach goes beyond the conventional focus on the effects of migration, as demonstrated by previous studies [24,113,114]. Our emphasis on empirical evidence, the vulnerability of targeted cities, and the integration of satellite images over the last 20 years adds depth to the literature, offering a more nuanced understanding of the causes and consequences of urbanization in Pakistan. It is the first ever study regarding the analysis and impact of socio-economic disparities, natural disasters and migration. First this study is conducted by targeting the most vulnerable cities in terms of rural to urban migration. We identified those factors empirically which have an impact on migration and confirmed the speculated statements about the causes of migration by providing the empirical evidence. Previous studies regarding these variable mainly focused on the effects of migration on socio-economic factors [84]. but this study not only identified the push factors from the place of origin and pull factors to place of residence but also suggest the policy implications to reduce the impact of migration on these major cities. Secondly we used satellite images of the last 20 years in order to get the true picture of the migrated population in the targeted cities, while previous studies in this regard were mainly confined to the growth of population while ignored the factors which are the real cause of the increased population in terms of migration [115,116].

To summarize, our results are firmly grounded in economic theories, supported by recent studies, and underscore the intricate relationship between socio-economic factors, natural disasters, and migration in the context of urbanization in Pakistan [4,39,46,117].

## 5. Conclusion

In conclusion, our comprehensive study on the impact of socio-economic disparities and natural disasters on migration from rural to urban areas and its subsequent effects on urbanization has yielded significant insights into these complex dynamics. The study reveals that both socio-economic disparities and natural disasters act as key drivers of rural-to-urban migration, with profound implications for urbanization. By integrating the principles of sustainable development, our research findings provide compelling evidence supporting the accepted hypotheses that both socio-economic disparities and natural disasters exert a positive influence on migration from rural to urban areas. Furthermore, our analysis reveals a significant positive relationship between migration and urbanization, demonstrating how migration drives urban growth and transformation. These findings highlight the interconnectedness of these phenomena and the need for sustainable policies to manage the impacts of this migration on urban areas.

The acceptance of the hypotheses underscores the critical role played by socio-economic factors and natural disasters in shaping rural to urban migration patterns. Socio-economic disparities, such as income inequality, limited access to education, and inadequate healthcare services, serve as major push factors that motivate rural populations to seek better opportunities in urban centers. Addressing these socio-economic disparities is essential not only for improving migration outcomes but also for achieving SDG 10 (Reducing Inequalities), which calls for more equitable economic opportunities. Similarly, the impact of natural disasters, ranging from floods to droughts and earthquakes, creates urgency and necessity for migration, as affected communities often relocate to urban areas seeking safety, stability, and resources to rebuild their lives. These insights suggest that addressing socio-economic inequalities and building climate resilience in rural areas is crucial for reducing migration pressures on cities. In light of SDG 11 (Sustainable Cities and Communities), the need for cities to be climate-resilient and capable of accommodating these migration flows is more critical than ever.

The positive relationship between migration and urbanization emphasizes the transformative effect of rural-to-urban migration on urban areas. As migrants move to urban centers, they contribute to the demographic, economic, and social fabric of cities. This influx of new residents not only diversifies the urban population but also presents opportunities for economic growth, cultural exchange, and innovation. However, this growth also poses challenges such as increased strain on urban infrastructure, housing, healthcare, and employment, which necessitate integrated urban planning that supports sustainable and inclusive growth. In order to realize the potential benefits of migration, urban planners and policymakers must work together to ensure that urban expansion is not only rapid but also sustainable, addressing the needs of both migrants and existing urban residents.

In light of these findings, policymakers, urban planners, and disaster management authorities must collaborate to develop holistic strategies that address the needs of both migrants and existing urban residents. Investments in education, healthcare, and skill development programs can empower migrants and enhance their socio-economic integration into urban communities. Additionally, urban infrastructure projects should be designed to accommodate the growing population, while environmental sustainability initiatives must be prioritized to mitigate the ecological impact of urbanization. These initiatives should align with global sustainability goals to ensure that cities can accommodate the increasing number of migrants without compromising the environment or quality of life.

In conclusion, our study underscores the intricate relationship between socio-economic disparities, natural disasters, rural to urban migration, and urbanization. By understanding these dynamics, societies can implement targeted policies that not only manage migration but also foster equitable, resilient, and sustainable urban growth. This approach will ensure that the process of rural-to-urban migration becomes a catalyst for positive change, enhancing the quality of life for all residents and promoting overall societal progress. Ultimately, fostering sustainable migration patterns and urbanization processes is essential to meet the challenges of the 21st century, contributing to long-term social stability and environmental sustainability.

### 5.1. Policy implications

#### 5.1.1. Addressing socio-economic disparities.

The positive correlation between socio-economic disparities and rural to urban migration underscores the need for targeted development initiatives in regions experiencing economic hardships. Government should allocate specific budget percentages for rural development initiatives based on needs assessments, prioritizing access to roads, transportation, schools, hospitals, electricity, and telecommunication in underserved regions. In line with sustainable development goals (SDGs), investments should also prioritize environmentally resilient infrastructure, renewable energy sources, and green job creation. Moreover, encourage public-private partnerships to attract investments in rural areas, offering tax breaks and incentives for companies that create jobs and provide essential services. Implement skill training programs tailored to rural needs, focusing on agriculture, entrepreneurship, and digital literacy to enhance economic opportunities as rural to urban migration can be tackled particularly in large cities like Karachi and Quetta.

Development agencies should design community-driven development projects that empower rural communities to identify their own infrastructure and service needs, fostering local ownership and sustainability. Additionally, supporting microfinance initiatives and small business development programs in rural areas to strengthen local economies and create diversified income streams, reducing the need for people to migrate to large cities like Karachi and Quetta.

#### 5.1.2. Mitigating natural disasters.

The significant correlation between natural disasters and migration calls for enhanced disaster preparedness and management strategies. Government, should invest in early warning systems, climate-resilient infrastructure, and evacuation plans to reduce the impact of natural disasters on vulnerable communities. Furthermore, disaster preparedness should incorporate climate adaptation strategies to ensure that both urban and rural communities can withstand future environmental shocks. Besides this must establish disaster preparedness training programs for local officials and communities, focusing on risk assessment, emergency response, and post-disaster recovery. Finally, develop relocation and resettlement plans for communities at high risk of displacement due to climate change and natural disasters, ensuring access to essential services and livelihoods in new locations instead of just letting them move to major cities that are already burdened.

There should be a role of NGOs in providing psychosocial support and livelihood assistance to disaster-affected communities, helping them rebuild their lives and livelihoods. Collaborate with government agencies to strengthen community-based disaster risk reduction initiatives, fostering local resilience.

#### 5.1.3. Managing urban growth.

The increase in urbanization observed in the GIS analysis emphasizes the need for sustainable urban planning and infrastructure development. Government must implement strict zoning regulations to prevent uncontrolled urban sprawl and protect environmentally sensitive areas. In line with SDG 11, investments in green infrastructure, sustainable public transport, and affordable housing are essential to reduce pollution, congestion, and strain on resources. As well as, invest in public transportation, affordable housing, and green infrastructure in cities to reduce pollution, congestion, and strain on resources, and prioritize the development of secondary cities and rural growth centers to decentralize economic opportunities and reduce migration pressure on major cities.

Lastly, the study is in line with previous studies [41,44,46] which also supports the push-pull theory of migration, and highlights the importance of socio-economic disparities and natural disasters as push factors for migration. Well directed government policies and investment in rural areas can help to manage the challenges of urbanization.

### 5.2. Limitations

While this study offers valuable insights, it has certain limitations to consider. The cross-sectional survey design limits our ability to establish causal relationships between variables. Additionally, the study focused on socio-economic disparities, natural disasters and migration as well as urban land growth (urbanization) specifically in cities, Karachi and Quetta, which may limit the generalizability of findings to other regions in Pakistan, however its potential applicability to other regions with shared characteristics, can open pathways for future research to validate findings in broader contexts. Future studies could also explore the environmental and climate-related aspects of migration, as well as the influence of sustainability policies on migration trends. Moreover, data on other potential contributing factors, such as government policies and climate change, were not collected, leaving room for further investigation.

## Supporting information

S1 File S1 Fig. Urbanization rate in Pakistan and the world, 1975–2050. S2 Fig. Snapshot of Natural Disasters for Last 10 years. S3 Fig. Area in sq. km of landcover of Karachi and Quetta city. **Note:** Supporting figures Fig 1, Fig 2, and Fig 7 from the main manuscript are presented here as Fig S1, Fig S2, and Fig S3, respectively.
(RAR)
